# Identification of bovine leukemia virus tax function associated with host cell transcription, signaling, stress response and immune response pathway by microarray-based gene expression analysis

**DOI:** 10.1186/1471-2164-13-121

**Published:** 2012-03-28

**Authors:** Mariluz Arainga, Eri Takeda, Yoko Aida

**Affiliations:** 1 Viral Infectious Diseases Unit, RIKEN, 2-1 Hirosawa, Wako, Saitama, 351-0198, Japan; 2 Laboratory of Viral Infectious Diseases, Department of Medical Genome Sciences, Graduate School of Frontier Science, The University of Tokyo, Wako, Saitama, 351-0198, Japan

## Abstract

**Background:**

Bovine leukemia virus (BLV) is associated with enzootic bovine leukosis and is closely related to human T-cell leukemia virus type I. The Tax protein of BLV is a transcriptional activator of viral replication and a key contributor to oncogenic potential. We previously identified interesting mutant forms of Tax with elevated (Tax_D247G_) or reduced (Tax_S240P_) transactivation effects on BLV replication and propagation. However, the effects of these mutations on functions other than transcriptional activation are unknown. In this study, to identify genes that play a role in the cascade of signal events regulated by wild-type and mutant Tax proteins, we used a large-scale host cell gene-profiling approach.

**Results:**

Using a microarray containing approximately 18,400 human mRNA transcripts, we found several alterations after the expression of Tax proteins in genes involved in many cellular functions such as transcription, signal transduction, cell growth, apoptosis, stress response, and immune response, indicating that Tax protein has multiple biological effects on various cellular environments. We also found that Tax_D247G_ strongly regulated more genes involved in transcription, signal transduction, and cell growth functions, contrary to Tax_S240P_, which regulated fewer genes. In addition, the expression of genes related to stress response significantly increased in the presence of Tax_S240P_ as compared to wild-type Tax and Tax_D247G_. By contrast, the largest group of downregulated genes was related to immune response, and the majority of these genes belonged to the interferon family. However, no significant difference in the expression level of downregulated genes was observed among the Tax proteins. Finally, the expression of important cellular factors obtained from the human microarray results were validated at the RNA and protein levels by real-time quantitative reverse transcription-polymerase chain reaction and western blotting, respectively, after transfecting Tax proteins into bovine cells and human HeLa cells.

**Conclusion:**

A comparative analysis of wild-type and mutant Tax proteins indicates that Tax protein exerts a significant impact on cellular functions as diverse as transcription, signal transduction, cell growth, stress response and immune response. Importantly, our study is the first report that shows the extent to which BLV Tax regulates the innate immune response.

## Background

Bovine leukemia virus (BLV), a retrovirus related to human T-cell leukemia virus types 1 and 2 (HTLV-1 and HTLV-2), causes enzootic bovine leukosis, a disease characterized by a very extended course that often involves persistent lymphocytosis (PL) and culminates in B-cell lymphoma [1]. BLV encodes the regulatory proteins Tax and Rex, which contribute to infectious potential and the regulation of viral expression [2,3]. The Tax protein acts on a triplicate 21-bp enhancer motif known as the Tax-responsive element (TxRE) in the U3 region of the 5^′^long terminal repeat (LTR), and it stimulates transactivation of the viral genome [2,4]. The TxRE consists of a cyclic AMP-response element (CRE)-like sequence, and Tax has been suggested to bind indirectly to this element through cellular factors, such as members of the CREB/activating transcription factor (ATF) family of basic leucine zipper proteins that have been shown to bind to the CRE-like sequence [5-7]. The Tax protein is also known to modulate the expression of cellular genes that are related to the regulation of cell growth [8]. Tax induces the immortalization of primary rat embryo fibroblasts and cooperates with the Ha-*ras* oncogene to induce the full transformation of cells that form tumors when injected into nude mice, a property shared by the G4 protein of BLV [9,10]. Furthermore, the expression of Tax in primary ovine B lymphocytes, which depends on CD154 and interleukin-4, affects B lymphocyte proliferation, cell cycle phase distribution, and survival, leading to cytokine-independent growth [11]. This immortalization process is also correlated with increased Bcl-2 protein levels, nuclear NF-κB accumulation, and a series of intracellular pathways that remain to be characterized [12]. In addition, Tax BLV inhibits base-excision DNA repair of oxidative damage, potentially increasing the accumulation of ambient mutations in cellular DNA [13]. To further understand the mechanisms of action, the identification of Tax-associated host cellular factors and pathways is now essential.

Our group has previously identified a Tax mutant in BLV that stimulates viral LTR-directed transcription via the BLV enhancer, and the extent of stimulation by this mutant is significantly greater than that of wild-type Tax and also that of a defective mutant that lacks this capacity [14]. The mutants have missense mutations in residues 247 (Tax_D247G_) and 240 (Tax_S240P_) that confer high and defective transactivation ability to the mutants, respectively [14]. Furthermore, it has been shown that Tax_D247G_ but not wild-type Tax protein activates the upstream sequence of the human cellular proto-oncogene c-*fos*, which contains 2 major *cis*-acting elements—the CArG box and CRE motif indispensable for the activation of *c-fos* by the mutant Tax protein—and also increases the levels of endogenous c- *fos* mRNA considerably in both human and bovine cell lines [8], suggesting that Tax_D247G_ might have the ability to activate the production of virus particles and an enhanced ability to induce leukemia/lymphosarcoma compared with wild-type Tax protein. Interestingly, we observed that an infectious molecular clone of BLV that encodes the Tax_D247G_ protein produced more viral particles and was transmitted at an elevated rate *in vitro*, but with no significant differences in the viral load and the expression of viral RNA between sheep experimentally injected with BLVs that encode the wild-type and mutant Tax_D247G_ proteins [15], suggesting a possible mechanism for regulating BLV–LTR–directed transcription by Tax that may play an important role in viral silencing *in vivo*. In addition, it appears that Tax protein has the capacity to participate in the induction and inhibition of cell survival *in vitro* and *ex vivo*, respectively, and mutant Tax protein caused greater stimulation than wild-type Tax protein did [16,17]. The enhanced activity of such a mutant Tax protein might be due to alterations in the interactions between Tax and cellular factors owing to the change in the amino acid sequence between amino acids 240 and 265. However, the mechanism by which such mutations increase the activity of Tax and the cellular factors involved in this process remains unknown.

The advent of microarray technology, coupled with the availability of comprehensive databases of gene identity and function, has made it possible to examine transcriptional changes across a landscape of thousands of genes. The human genome is almost fully annotated, and thus, chips representing all of the annotated genes are commercially available. By contrast, the quality of commercially and custom-made genome-wide microarrays is still poor because of the limited availability of full-length cDNA sequences in cattle. In this study, in an attempt to identify genes that play a role in the cascade of signal events regulated by wild-type Tax (Tax_WT_) and mutant Tax with high (Tax_D247G_) and defective transactivation activity (Tax_S240P_), we used a large-scale host cell gene-profiling approach using human cDNA microarrays. Furthermore, we chose human HeLa cells, which have been extensively characterized regarding the host factors involved in various cellular functions as a model system for analysis of bovine Tax proteins; isolated total RNA from HeLa cells had been transfected with expression vectors encoding Tax_WT_, Tax_S240P_, Tax_D247G_, or control vector; and subjected each RNA sample to human microarray analysis. The genomic expression profiling resulted in the identification of approximately 300 genes up- and downregulated by wild-type and mutant Tax proteins. Finally, the expression of important transcriptional cellular factors indicated by the human microarray results were validated after the transfection of wild-type or mutant Tax proteins into bovine cells as well as human HeLa cells.

## Results

### Expression of wild-type and mutant tax proteins in HeLa cells

**Figure 1:**
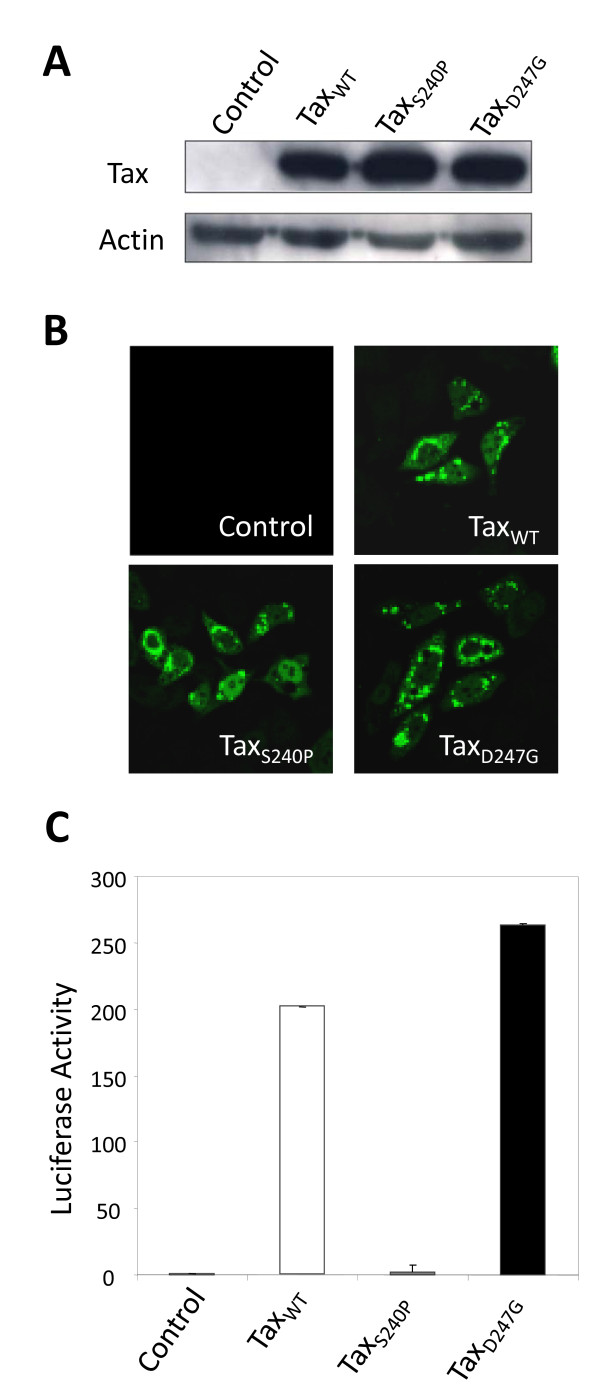
**Expression, localization, and transactivation of wild-type and mutant Tax proteins in HeLa cells.** HeLa cells were transfected with pCAGGS encoding Flag-tagged Tax proteins (Tax_WT_, Tax_S240P_, or Tax_D247G_) or the control pCAGGS without (**A** and **B**) or with the reporter plasmid pGV-BLTR and the reference plasmid pRL-SV40 ( **C**). Thereafter, 24 h after transfection, cells were subjected to western blotting ( **A**), confocal laser-scanning analysis ( **B**), and the firefly luciferase assay ( **C**). ( **A**) Western blotting was performed using an anti-Flag M2 MAb and anti-actin antibody as a control. ( **B**) For immunofluorescence, cells were fixed, permeabilized, and immunostained with anti-Flag M2 MAb followed by the Alexa-488-conjugated anti-mouse antibody (green). ( **C**) For the transactivation assay, cells were recovered and lysed, and the activities of firefly and *Renilla* luciferase were measured. For each sample, the firefly luciferase activity (pGV-BLTR) was normalized by reference to *Renilla* luciferase activity (pRL-SV40). Average values from triplicate transfections with standard deviations (error bars) are shown (*p < 0.01).

To measure alterations in the expression of host cellular genes regulated by wild-type and mutant Tax proteins with elevated (D247G) and reduced (S240P) transactivation activity, we constructed pCAGGS mammalian expression vectors encoding Tax proteins (Tax_WT_, Tax_S240P_, and Tax_D247G_) that were Flag-tagged at their carboxy termini to facilitate the assay, and these vectors were transfected into human cervical HeLa cells. At 24 h after transfection, we examined the expression of each Tax protein by western blotting using monoclonal antibody (MAb) M2, which recognizes the Flag tag (Figure 1A). We observed a single band for each of the 3 vector constructs, and found no difference in protein expression between wild-type and mutant Tax proteins in repeated experiments. As shown in Figure 1B, all Tax proteins exhibited greater cytoplasmic localization than nuclear localization and exhibited punctate staining patterns in the majority of transfected HeLa cells, as previously described by [18]. To clarify the transactivation capacity of mutant Tax proteins, we transfected HeLa cells with Tax expression vectors plus the reporter plasmid pGV-BLTR, which expressed the full-length BLV LTR upstream of the luciferase gene, and then, we analyzed the luciferase activity (Figure 1C). Tax_D247G_ was able to induce higher viral LTR-directed transcriptional activation than the Tax_WT_, in contrast to Tax_S240P_, which markedly reduced this activity, in line with the result of our previous study using 293 T cells [14]. Thus, we confirmed that there was no obvious relationship between transactivation activity and the expression, stability, and localization of Tax proteins in HeLa cells. Therefore, we used HeLa cells in subsequent microarray experiments.

### Large-scale expression profiling of cellular genes after the transfection of wild-type and mutant tax proteins

**Figure 2:**
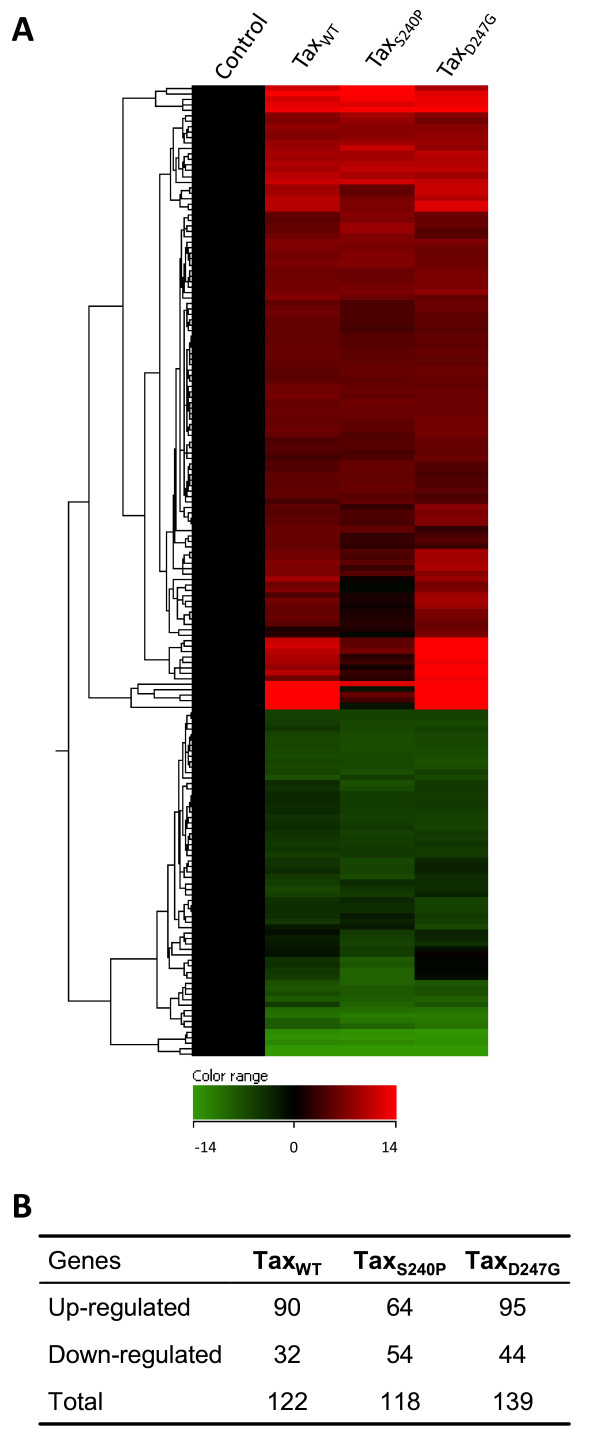
**Differential expression of cellular genes after induction of wild-type and mutant Tax proteins in HeLa cells.** Total RNA from HeLa cells transfected with pCAGGS encoding Tax proteins (Tax_WT_, Tax_S240P_, or Tax_D247G_) or the control pCAGGS was used for the microarray analysis. (**A**) Heat map of hierarchical gene clustering. The microarray data was analyzed using GeneSpring GX 11.0 software to obtain the hierarchical clustering. The columns correspond to gene profiles regulated by the different Tax proteins indicated above the map. Below the heat map is a color-coded scale bar for the relative expression levels of genes. ( **B**) Number of genes significantly up- or downregulated by wild-type and mutant Tax proteins. The genes shown in this figure passed 2 filtering criteria: (1) statistical significance (p < 0.05) and (ii) at least 2-fold changes in the expression of a cellular gene at least 1 time point.

To analyze the transcriptional effects by wild-type and mutant Tax proteins on global gene expression, total RNA was isolated from HeLa cells that had been transfected with Tax_WT_, Tax_S240P_, Tax_D247G_, or control vector, and each RNA sample was subjected to microarray analysis. Additionally, to ensure equivalent transfection efficiency, we simultaneously assessed the expression of all Tax proteins and confirmed same efficiency in our samples (data not shown). After microarray analysis, data sets were analyzed using GeneSpring GX 11.0 software for gene expression, clustering, gene ontology, and significant pathway signaling. Using DNA microarrays containing approximately 18,400 mRNA transcripts in each array, we identified over 300 genes hierarchically clustered for all samples (Figure 2A). Among them, 122 (90 were upregulated and 32 were downregulated), 118 (64 were upregulated and 54 were downregulated), and 139 genes (95 were upregulated and 44 were downregulated) exhibited statistically significant differential regulation (2-fold or more) by Tax_WT_, Tax_S240P_, and Tax_D247G_, respectively (p < 0.05) (Figure 2B). The mutant Tax_S240P_ induced more variation in whole gene expression, as noted by the colors in the heat map (Figure 2A), which indicated that this mutant upregulated (red) fewer genes and downregulated (blue) more genes than the other Tax proteins did. The numbers of genes up- and downregulated by Tax_WT_ or Tax_D247G_ were similar, but the intensity of the expression changes was slightly stronger in the case of Tax_D247G_.

**Figure 3:**
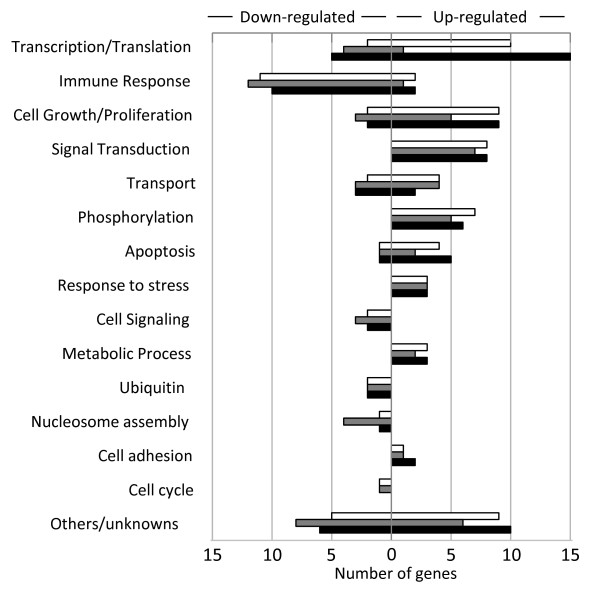
**Ontology annotations of differentially expressed genes after the transfection of wild-type and mutant Tax proteins.** Classification of up- or downregulated genes involved in biological functions with significant differential expression (fold change > 2.0 and p < 0.05). Tax_WT,_ open column; Tax_S240_, shaded column; Tax_D247G_, black column.

Analysis of gene ontology annotations revealed the number of genes grouped according to biological functions that were upregulated by wild-type and mutant Tax proteins by at least 2-fold (p < 0.05) (Figure 3). The upregulated genes were clustered within functional groups involved in transcription/translation/RNA processing, cell growth/proliferation, signal transduction, transport, phosphorylation, apoptosis, stress response, metabolic processes, and cell adhesion. Interestingly, we found that Tax_D247G_ regulated more genes involved in transcription/translation/RNA processing functions, contrary to Tax_S240P_, which regulated fewer genes (Figure 3). As shown in Table 1, Tax_D247G_ induced higher expression for the following genes than Tax_WT_ and Tax_S240P_ did: genes involved in transcription/translation/RNA processing such as CREM, FOS, NR4A2, RORA*,* MAFB, and MAFF; genes involved in signal transduction such as GEM, GNB5, PITPNC1, RGS5, and RRAD; genes involved in cell growth/cell proliferation such as CYR61, EMP3, and IL11; TNFAIP6, which is involved in immune responses; NPTX1, which is involved in transport; PHLDA1, which is involved in apoptosis; and DUSP1, which is a dual specificity serine/threonine phosphatase. All these genes were expressed at lower level in cells transfected with Tax_S240P_, which indicated the reduced transactivation activity of Tax_S240P_ compared to that of Tax_WT_ or Tax_D247G_. By contrast, the expression of DNAJB1, HSPA1A, and HSPA6, which respond to stress, was significantly increased in the presence of Tax_S240P_ compared to that in the presence of the other Tax proteins. There were no differences in the expression levels of genes related to apoptosis, such as IER3, TNFRSF12A, and NFRSF21; genes related to transcription/translation/RNA processing, such as ETV5 and JUN; and genes related to signal transduction, such as GDF15, induced by the Tax proteins. Thus, we confirmed that our mutant Tax_D247G_ strongly induced the expression of cellular factors involved in transcription/translation/RNA processing, signal transduction, and cell growth/cell proliferation, whereas the mutant Tax_S240P_ could not induce these genes at high expression levels. Our result also shows that the mutant Tax_S240P_ upregulated genes related to stress responses.

**Table 1:** Genes upregulated in the total RNA fraction in the presence of wild-type and mutant Tax proteins

**Gene ID**	**Gene description**	**Gene Symbol**	**Fold Change**		
			**Tax**_**WT**_	**Tax**_**S240P**_	**Tax**_**D24G**_
	**Transcription/Translation/RNA processing**				
1390	cAMP responsive element modulator	CREM	2.2	1.8	3.2
1958	early growth response 1	EGR1	1.9	2.0	1.7
22936	elongation factor, RNA polymerase II, 2	ELL2	2.2	2.2	2.2
2119	ets variant 5	ETV5	3.7	3.6	3.4
2353	FBJ murine osteosarcoma viral oncogene homolog	FOS	3.7	2.4	4.6
8061	FOS-like antigen 1	FOSL1	2.4	2.4	2.3
3280	Hairy and enhancer of split 1 (Drosophila)	HES1	1.8	1.9	2.0
3725	jun oncogene	JUN	4.7	5.9	5.3
10365	Kruppel-like factor 2 (lung)	KLF2	2.6	2.6	2.8
1316	Kruppel-like factor 6	KLF6	2.1	2.1	2.1
9935	v-maf musculoaponeurotic fibrosarcoma oncogene homolog B (avian)	MAFB	2.6	1.9	3.8
23764	v-maf musculoaponeurotic fibrosarcoma oncogene homolog F (avian)	MAFF	2.4	1.9	2.6
4929	nuclear receptor subfamily 4, group A, member 2	NR4A2	3.6	1.5	7.7
57157	Putative homeodomain transcription factor 2	PHTF2	1.9	1.9	2.0
6095	RAR-related orphan receptor A	RORA	10.3	2.0	10.6
7538	zinc finger protein 36, C3H type, homolog (mouse)	ZFP36	2.8	2.6	2.6
	**Signal Transduction**				
287	ankyrin 2, neuronal	ANK2	2.0	2.0	1.4
9518	growth differentiation factor 15	GDF15	5.0	4.9	5.6
2669	GTP binding protein over expressed in skeletal muscle	GEM	14.3	5.0	19.1
10681	guanine nucleotide binding protein (G protein), beta 5	GNB5	2.0	1.2	2.4
2786	guanine nucleotide binding protein (G protein), gamma 4	GNG4	1.8	2.0	1.6
26207	phosphatidylinositol transfer protein, cytoplasmic 1	PITPNC1	1.9	1.7	2.5
5329	plasminogen activator, urokinase receptor	PLAUR	2.0	1.7	1.9
5521	protein phosphatase 2 (formerly 2A), regulatory subunit B, beta isoform	PPP2R2B	1.3	1.0	2.1
5997	regulator of G-protein signaling 2, 24 kDa	RGS2	2.4	1.1	2.4
6004	regulator of G-protein signaling 16	RGS16	2.0	2.0	2.0
6236	Ras-related associated with diabetes	RRAD	3.8	2.3	7.9
81848	sprouty homolog 4 (Drosophila)	SPRY4	1.9	2.1	1.9
	**Inflammatory response/Immune response**				
2921	chemokine (C-X-C motif) ligand 3	CXCL3	1.8	2.0	1.8
3269	histamine receptor H1	HRH1	2.5	1.7	1.8
7130	tumor necrosis factor, alpha-induced protein 6	TNFAIP6	20.5	1.3	36.6
	**Regulation of cell growth/Regulation of cell proliferation**				
183	angiotensinogen (serpin peptidase inhibitor, clade A, member 8)	AGT	1.3	1.3	2.3
374	amphiregulin	AREG	2.0	2.1	2.3
694	B-cell translocation gene 1, anti-proliferative	BTG1	3.3	1.1	3.0
6347	chemokine (C-C motif) ligand 2	CCL2	2.0	1.6	1.6
960	CD44 molecule (Indian blood group)	CD44	2.1	2.0	2.3
3491	cysteine-rich, angiogenic inducer, 61	CYR61	4.8	2.0	7.5
2014	epithelial membrane protein 3	EMP3	1.8	1.2	3.1
3589	interleukin 11	IL11	3.6	1.7	5.4
83729	inhibin, beta E	INHBE	2.1	3.0	1.9
182	jagged 1	JAG1	2.1	1.8	2.3
3976	leukemia inhibitory factor (cholinergic differentiation factor)	LIF	2.2	2.2	2.2
	**Apoptosis**				
56892	chromosome 8 open reading frame 4	C8orf4	1.2	1.2	2.3
8870	immediate early response 3	IER3	3.3	3.2	3.7
22822	pleckstrin homology-like domain, family A, member 1	PHLDA1	2.5	1.9	3.2
51330	tumor necrosis factor receptor superfamily, member 12A	TNFRSF12A	2.0	1.9	2.0
27242	tumor necrosis factor receptor superfamily, member 21	TNFRSF21	2.3	2.1	2.4
	**Cell adhesion/Cell-cell adhesion**				
6695	sparc/osteonectin, cwcv and kazal-like domains proteoglycan 1	SPOCK1	2.0	1.7	2.1
6696	secreted phosphoprotein 1	SPP1	1.9	2.5	2.1
	**Transport**				
3039	hemoglobin, alpha 1	HBA1	2.4	2.5	2.4
4884	neuronal pentraxin I	NPTX1	2.3	1.2	3.6
6507	solute carrier family 1, member 3	SLC1A3	1.8	2.0	1.8
6533	solute carrier family 6, member 6	SLC6A6	2.2	2.3	1.9
23657	solute carrier family 7, member 11	SLC7A11	2.3	2.4	1.8
	**Metabolic process**				
384	arginase, type II	ARG2	3.2	3.2	3.6
9945	glutamine-fructose-6-phosphate transaminase 2	GFPT2	2.0	2.0	1.9
9388	Lipase, endothelial	LIPG	1.9	1.4	2.1
7378	uridine phosphorylase 1	UPP1	2.1	1.8	2.0
	**Phosphorylation/Dephosphorylation**				
9201	doublecortin-like kinase 1	DCLK1	2.1	2.0	2.1
1843	dual specificity phosphatase 1	DUSP1	3.6	2.4	4.7
51207	dual specificity phosphatase 13	DUSP13	2.2	1.9	2.2
1846	dual specificity phosphatase 4	DUSP4	2.9	3.0	2.9
1847	dual specificity phosphatase 5	DUSP5	2.4	2.4	2.8
1848	dual specificity phosphatase 6	DUSP6	2.4	2.8	2.7
5578	protein kinase C, alpha	PRKCA	2.1	1.9	1.8
	**Response to stress**				
3337	DnaJ (Hsp40) homolog, subfamily B, member 1	DNAJB1	2.7	3.1	2.5
3303	heat shock 70 kDa protein 1A	HSPA1A	2.9	4.2	2.8
3310	heat shock 70 kDa protein 6	HSPA6	6.4	15.4	5.3
	**Others/Unknown**				
202	absent in melanoma 1	AIM1	1.7	1.8	2.1
23237	activity-regulated cytoskeleton-associated protein	ARC	2.5	2.3	2.3
1475	cystatin A (stefin A)	CSTA	4.6	5.3	5.4
56603	cytochrome P450, family 26, subfamily B, polypeptide 1	CYP26B1	2.1	1.7	2.1
23052	endonuclease domain containing 1	ENDOD1	2.0	2.1	2.1
2201	fibrillin 2	FBN2	2.1	1.6	1.8
10324	kelch repeat and BTB (POZ) domain containing 10	KBTBD10	3.0	1.9	2.4
5270	serpin peptidase inhibitor, clade E, member 2	SERPINE2	2.3	2.3	2.4
4071	transmembrane 4 L six family member 1	TM4SF1	1.9	1.8	2.3
25907	transmembrane protein 158 (gene/pseudogene)	TMEM158	2.1	2.4	2.5
7171	tropomysin 4	TPM4	2.5	1.5	3.5
7846	tubulin, alpha 1a	TUBA1A	1.9	2.1	1.8

We next assessed whether the expression of wild-type and mutant Tax proteins resulted in the transcriptional downregulation of any cellular transcripts. The downregulated genes were involved in transcription/translation/RNA processing, immune response, cell growth/proliferation, apoptosis, cell cycle, transport, cell signaling, ubiquitination, and nucleosome assembly (Figure 3). In particular, a number of molecules involved in immune response were significantly downregulated by all of the Tax proteins. As shown in Table 2, we found 12 downregulated genes involved in immune response and the immune response to virus infection, and interestingly, they were mainly related to the interferon family of anti-viral factors, such as IFIT1, IFIT3, and OASL. In general, the expression levels of the downregulated genes were similarly affected by the 3 Tax proteins. Thus, our results clearly demonstrated a significant negative impact of Tax on expression of genes involved in the innate immune response to viral infection.

**Table 2:** Genes downregulated in the total RNA fraction in the presence of wild-type and mutant Tax proteins

**Gene ID**	**Gene description**	**Gene Symbol**	**Fold Change**		
			**Tax**_**WT**_	**Tax**_**S240P**_	**Tax**_**D247G**_
	**Transcription/Translation/RNA processing**				
23741	EP300 interacting inhibitor of differentiation 1	EID1	2.0	2.1	2.0
2625	GATA binding protein 3	GATA3	2.0	1.6	1.6
3398	inhibitor of DNA binding 2, dominant negative helix-loop-helix protein	ID2	1.6	1.4	2.2
23658	LSM5 homolog, U6 small nuclear RNA associated (S. cerevisiae)	LSM5	1.7	1.8	2.0
63931	mitochondrial ribosomal protein S14	MRPS14	1.9	2.4	2.2
5936	RNA binding motif protein 4	RBM4	1.7	2.4	2.1
6591	snail homolog 2 (Drosophila)	SNAI2	1.9	2.2	1.7
	**Immune response/Response to virus**				
23586	DEAD (Asp-Glu-Ala-Asp) box polypeptide 58	DDX58	2.9	3.3	3.9
79132	DEXH (Asp-Glu-X-His) box polypeptide 58	DHX58	2.1	2.2	2.3
10561	interferon-induced protein 44	IFI44	2.1	2.7	2.8
64135	interferon induced with helicase C domain 1	IFIH1	2.0	2.2	2.3
3434	interferon-induced protein with tetratricopeptide repeats 1	IFIT1	5.2	5.6	6.0
3433	interferon-induced protein with tetratricopeptide repeats 2	IFIT2	3.1	3.7	4.2
3437	interferon-induced protein with tetratricopeptide repeats 3	IFIT3	5.4	5.5	5.5
9636	ISG15 ubiquitin-like modifier	ISG15	2.2	2.6	2.1
8638	2'-5'-oligoadenylate synthetase-like	OASL	8.8	8.8	10.3
445347	TCR gamma alternate reading frame protein	TARP	1.9	3.0	1.1
6967	T cell receptor gamma constant 2	TRGC2	2.1	3.4	1.2
56829	zinc finger CCCH-type, antiviral 1	ZC3HAV1	2.9	2.9	3.2
	**Cell signalling**				
3641	insulin-like 4 (placenta)	INSL4	2.4	2.7	2.5
5122	proprotein convertase subtilisin/kexin type 1	PCSK1	2.8	2.8	2.5
25780	RAS guanyl releasing protein 3 (calcium and DAG-regulated)	RASGRP3	1.6	2.1	1.8
	**Nucleosome assembly**				
94239	H2A histone family, member V	H2AFV	1.9	2.0	1.9
8334	histone cluster 1, H2ac	HIST1H2AC	2.1	2.0	2.0
8329	histone cluster 1, H2ag	HIST1H2AG	1.6	2.2	1.7
8349	histone cluster 2, H2be	HIST2H2BE	1.7	2.2	1.6
	**Regulation of cell proliferation**				
2247	fibroblast growth factor 2 (basic)	FGF2	3.4	3.5	3.7
10216	proteoglycan 4	PRG4	1.7	2.2	1.7
5920	retinoic acid receptor responder (tazarotene induced) 3	RARRES3	2.3	2.4	2.5
	**Apoptosis**				
8743	tumor necrosis factor (ligand) superfamily, member 10	TNFSF10	2.5	2.9	2.6
	**Cell cycle**				
10083	Usher syndrome 1 C (autosomal recessive, severe)	USH1C	2.0	2.0	1.7
	**Transport**				
57101	anoctamin 2	ANO2	1.9	2.1	2.0
6337	sodium channel, nonvoltage-gated 1 alpha	SCNN1A	2.4	2.1	2.4
6717	sorcin	SRI	2.3	2.3	2.6
	**Ubiquitin**				
51191	hect domain and RLD 5	HERC5	4.9	5.0	5.7
55008	hect domain and RLD 6	HERC6	2.2	2.5	2.2
	**Others/Unknown**				
83990	BRCA1 interacting protein C-terminal helicase 1	BRIP1	1.4	2.1	1.8
79940	chromosome 6 open reading frame 155	C6orf155	1.5	2.1	1.5
1601	disabled homolog 2, mitogen-responsive phosphoprotein (Drosophila)	DAB2	2.2	2.3	2.2
55601	DEAD (Asp-Glu-Ala-Asp) box polypeptide 60	DDX60	3.7	4.1	4.3
2335	fibronectin 1	FN1	2.0	2.5	1.8
23387	SIK family kinase 3	KIAA0999	1.2	1.2	2.2
54809	sterile alpha motif domain containing 9	SAMD9	1.8	2.0	2.4
6414	selenoprotein P, plasma, 1	SEPP1	2.1	2.1	2.2
8470	sorbin and SH3 domain containing 2	SORBS2	2.2	2.4	2.4

Moreover, we determined which biological pathways and genes involved in these pathways were significantly affected by wild-type and mutant Tax proteins (p < 0.05) (Table 3). Almost all the pathways were represented by the same genes regulated by all of our Tax constructs, and these pathways and genes are involved in many transcriptional functions, such as p53 effectors, transcriptional targets of AP1 family, IL1-mediated signaling, regulation of p38 MAPK signaling, IL12-mediated signaling, IL2-mediated signaling, ATF-2 transcription factor network, JNK signaling in the CD4+ TCR pathway, and Ras signaling in the CD4+ TCR pathway (Table 3).

**Table 3:** Genes involved in signaling pathways regulated by wild-type and mutant Tax proteins (p < 0.05)*

**Significant Pathway**	**Tax**_**WT**_	**Tax**_**S240P**_	**Tax**_**D247G**_
Direct p53 effectors	DUSP1, DUSP5, GDF15, HSPA1A, JUN, LIF	*BDKRB2*, DUSP1, DUSP5, GDF15, HSPA1A, JUN, LIF, *SNAI2*, SPP1	DUSP1, DUSP5, GDF15, HSPA1A, JUN, LIF, SPP1
Validated transcriptional targets of AP1 family members Fra1 and Fra2	FOS, FOSL1, GATA3, JUN, LIF, PLAUR	FOS, FOSL1, IL8, JUN, LIF	FOS, FOSL1, JUN, LIF
IL1-mediated signaling events	DUSP1, FOS, FOSL1, *GATA3*, JUN	DUSP1, FOS, FOSL1, IL8, JUN	DUSP1, FOS, FOSL1, JUN
BMP receptor signalling	DUSP1, FOS, FOSL1, *GATA3*, JUN	DUSP1, FOS, FOSL1, IL8, JUN	DUSP1, FOS, FOSL1, JUN
p38 MAPK signaling pathway	DUSP1, FOS, FOSL1, *GATA3*, JUN	DUSP1, FOS, FOSL1, IL8, JUN	DUSP1, FOS, FOSL1, JUN
Regulation of p38-alpha and p38-beta	DUSP1, FOS, FOSL1, *GATA3*, JUN	DUSP1, FOS, FOSL1, IL8, JUN	DUSP1, FOS, FOSL1, JUN
IL12-mediated signaling events	ETV5, FOS, *GATA3*, JUN	ETV5, FOS, IL12A, JUN	ETV5, FOS, JUN
Role of Calcineurin-dependent NFAT signaling in lymphocytes	FOS, FOSL1, *GATA3*, JUN	FOS, FOSL1, IL8, JUN	FOS, FOSL1, JUN
Calcineurin-regulated NFAT-dependent transcription in lymphocytes	FOS, FOSL1, *GATA3*, JUN	FOS, FOSL1, IL8, JUN	FOS, FOSL1, JUN
FOXM1 transcription factor network	ETV5, FOS, HSPA1A	ETV5, FOS, HSPA1A	ETV5, FOS, HSPA1A
xef;ve CD4+ T cells	FOS, FOSL1, JUN	FOS, FOSL1, JUN	FOS, FOSL1, JUN
CXCR4-mediated signaling events	FOS, FOSL1, JUN	FOS, FOSL1, JUN	FOS, FOSL1, JUN
Glucocorticoid receptor signaling	FOS, *GATA3*, JUN	FOS, IL8, JUN	
ATF-2 transcription factor network	DUSP1, DUSP5, JUN	DUSP1, DUSP5, IL8, JUN	DUSP1, DUSP5, HES1, JUN
JNK signaling in the CD4+ TCR pathway	FOSL1, JUN	FOS, FOSL1, JUN	FOS, FOSL1, JUN
Ras signaling in the CD4+ TCR pathway	FOS, FOSL1, JUN	FOS, FOSL1, JUN	FOS, FOSL1, JUN
xef;ve CD8+ T cells	FOS, FOSL1, JUN	FOS, FOSL1, JUN	FOS, FOSL1, JUN
Calcium signaling in the CD4+ TCR pathway	FOS, FOSL1, JUN	FOS, FOSL1, JUN	FOS, FOSL1, JUN
Regulation of Wnt-mediated beta catenin signaling and target gene transcription	CYR61, JUN	IL8, JUN, *SNAI2*	CYR61, *ID2*, JUN
IL2-mediated signaling events	FOS, JUN	FOS, JUN	FOS, JUN
ErbB2/ErbB3 signaling events	FOS JUN	FOS, JUN	FOS, JUN
BCR signaling pathway	FOS, JUN	FOS, JUN	FOS, JUN
IL12 signaling mediated by STAT4	ETV5, JUN	ETV5, JUN	ETV5, JUN
PDGFR-alpha signaling pathway	FOS, JUN	FOS, JUN	FOS, JUN
Integrins in angiogenesis	CD44, *FGF2*	CD44, *FGF2*, SPP1	CD44, *FGF2*, SPP1
amb2 Integrin signalling	CYR61, PLAUR		

*Up- or downregulated genes are represented by regular or italic characters, respectively.

### Validation of the expression of host cellular genes at the RNA and protein levels in HeLa cells

**Figure 4:**
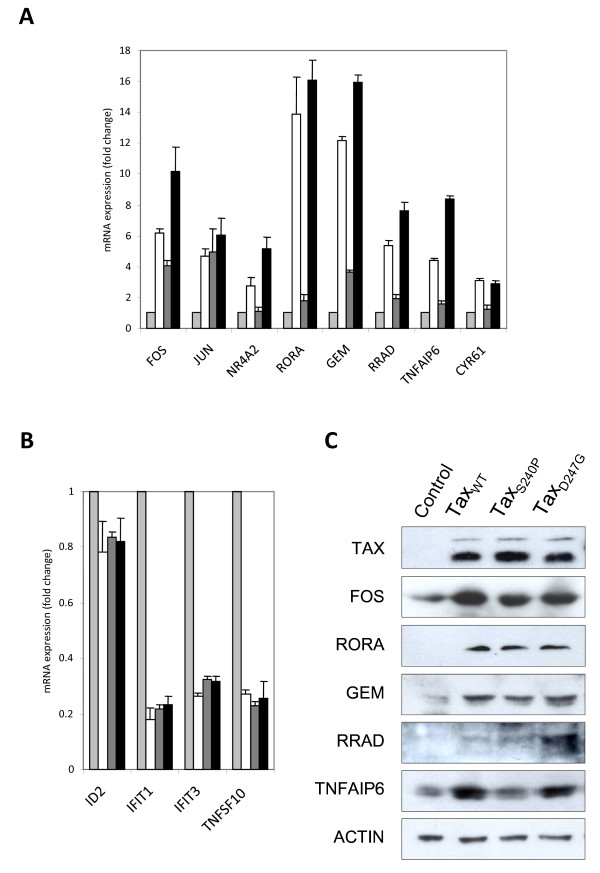
**Validation of differentially expressed genes at the RNA and protein levels in HeLa cells.** Differential expression of up- ( **A**) or downregulated ( **B**) genes in HeLa cells transfected with pCAGGS encoding Flag-tagged Tax proteins (Tax_WT,_ open column; Tax_S240_, shaded column; Tax_D247G_, black column) or the control pCAGGS (light column) was validated by real-time RT-PCR. The bars indicate the fold change expression of the genes regulated by Tax. Data were normalized to GAPDH mRNA expression. The results represent the mean of 2 samples from 1 experiment. (**C**) Protein expressions of Tax, Fos, Rora, Rrad, Gem, and Tnfaip6 were validated by western blotting. Actin was used as a control.

To validate the microarray data after overexpression of wild-type and mutant Tax proteins in HeLa cells, we assessed the expression of several transcriptional cellular factors at the RNA and protein levels (Figure 4). First, real-time quantitative reverse transcription-polymerase chain reaction (qRT-PCR) was used to corroborate the fold changes obtained from the microarray results. In performing this assay, we chose significantly upregulated genes in 4 categories: transcription/translation/RNA processing (FOS, JUN, NR4A2, and RORA), signal transduction (GEM and RRAD), immune response (TNFAIP6), and regulation of cell growth and cell proliferation (CYR61). The qRT-PCR results for these genes in HeLa cells that had been transfected with pCAGGS encoding Tax_WT_, Tax_S240P_, or Tax_D247G,_ are presented in Figure 4. Representative qRT-PCR data were consistent with the data of microarrays in 7 validated cases (FOS, NR4A2, RORA, GEM, RRAD, TNFAIP6, and CYR61) in which Tax_D247G_ induced higher gene expression than Tax_WT_ and Tax_S240P_ did and in 1 validated case (JUN) in which the gene expression was similar for all 3 Tax proteins. However, the fold expression of the TNFAIP6 gene observed by the microarray analysis was higher than that in the qRT-PCR analysis. For instance, according to the microarray analysis, TNFAIP6 expression was upregulated 20.5-, 1.3-, and 36.6-fold in the presence of Tax_WT_, Tax_S240P_, and Tax_D247G_, respectively, compared with 5-, 2-, and 8-fold according to qRT-PCR. These qRT-PCR results confirmed that Tax_D247G_ induced strong responses of genes, whereas Tax_S240P_ induced the weakest responses among the 3 Tax proteins.

We next validated the results of the genes that were downregulated in Tax-expressing HeLa cells. For this purpose, we selected genes related to transcription/translation/RNA processing (ID2), immune response/response to virus (IFIT1 and IFIT3), and apoptosis (TNFSF10). As shown in Figure 4B, the expression of all of these genes were very low in the presence of Tax proteins, and there were no differences in the expression of these genes in the presence of wild-type and mutant Tax proteins, which perfectly correlated with our microarray results.

Finally, to further confirm that the changes observed at the RNA level were consistent at the protein level, we used western blotting to determine the expression of FOS, RORA, GEM, RRAD, TNFAIP6, and Actin as a control (Figure 4C). The results from this analysis indicate that Tax induced the overexpression of FOS, RORA, GEM, RRAD, and TNFAIP6; however, in the case of FOS, GEM, and TNFAIP6, the expression was higher in the presence of Tax_WT_ or Tax_D247G_ than in the presence of Tax_S240P_. However, Tax_S240P_ did not induce the overexpression of TNFAIP6. RRAD was highly overexpressed in the presence of Tax_D247G_, but there was no difference between the expression induced by Tax_WT_ and that induced by Tax_S240P_. By contrast, all of our Tax proteins induced similar levels of RORA expression. In general, all of these results were in agreement with the microarray results in HeLa cells, supporting the authenticity of our microarray data.

### Validation of the expression of host cellular genes at the RNA and protein levels in bovine 23CLN cells

**Figure 5:**
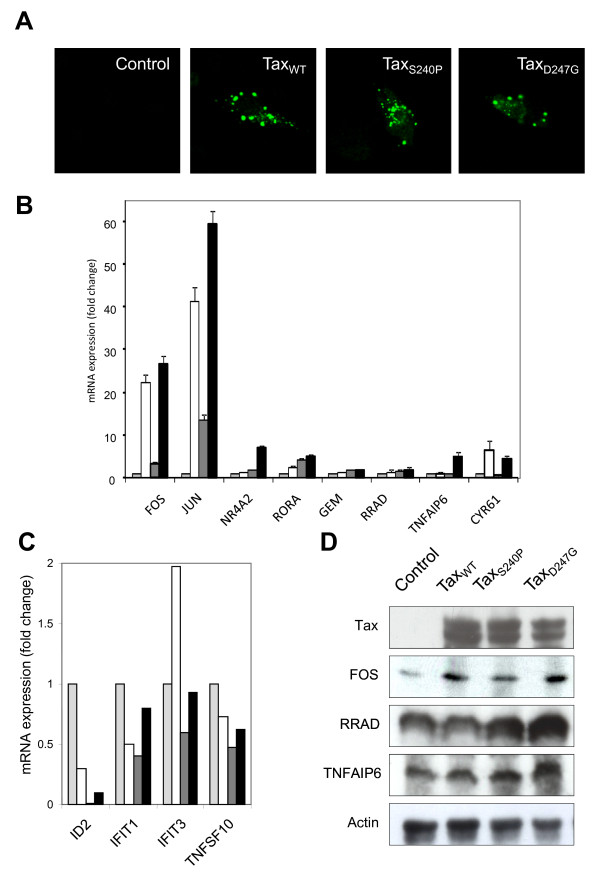
**Validation of differentially expressed genes at the RNA and protein levels in bovine 23CLN cells.** 23CLN cells were transfected with pCAGGS encoding Flag-tagged Tax proteins (Tax_WT,_ open column; Tax_S240_, shaded column; Tax_D247G_, black column) or the control pCAGGS (light column). Thereafter, 24 h after transfection, cells were subjected to confocal laser-scanning analysis to detect localization (**A**) and to real-time RT-PCR ( **B** and **C**) and western blotting ( **D**) to validate in bovine cells differential expression of cellular genes had altered after expression of wild-type and mutant Tax proteins in HeLa cells. The bars indicate the fold change expression of up- ( **B**) or downregulated ( **C**) genes regulated by Tax. Data were normalized to GAPDH mRNA expression. The results represent the mean of 2 samples from 1 experiment. ( **D**) The protein expression of Tax and Fos, Rrad, and Tnfaip6 was validated by western blotting. Actin was used as a control.

Because our microarray analysis was performed in human cells and human arrays, we determined whether similar results could be obtained in bovine cells, the natural host for BLV. Therefore, we used bovine lymph node 23CLN cells for the purpose of this study. First, to detect protein expression and localization in 23CLN cells, pCAGGS vectors encoding Tax proteins were transfected into 23CLN cells, and then, the cells were subjected to confocal laser-scanning analysis and western blotting. As shown in Figure 5A, all 3 proteins localized predominantly to the cytoplasm, with lesser amounts detected in the nucleus, as observed for HeLa cells (Figure 1A). Western blotting analysis with the MAb M2 revealed that each Tax protein was expressed at detectable levels in the corresponding transfected 23CLN cells (Figure 5D). Moreover, Tax_WT_ and Tax_D247G_ were able to induce higher viral LTR-directed transcriptional activation than Tax_S240P_ (data not shown), similarly as observed in HeLa cells.

Later, we examined in Tax-expressing bovine cells the expression levels of factors, FOS, JUN, NR4A2, RORA, GEM, RRAD, TNFAIP6, and CYR61 genes, confirmed as being upregulated in Tax-expressing HeLa cells by qRT-PCR in Figure 4A (Figure 5B). It is important to mention that FOS and JUN were highly overexpressed in bovine cells compared to its expression in human cells. By contrast, it should also be noted that RORA, GEM, RRAD and TNFAIP6 genes which showed significantly high gene expression in human cells, were only slightly upregulated in 23CLN cells. Furthermore, four genes, ID2, IFIT1, IFIT3 and TNFSF10, were downregulated in the presence of Tax in 23CLN cells in accordance to our microarray data in HeLa cells; but surprisingly, only Tax_WT_ up-regulated the expression of IFIT3 with the two-hold increase as compared to the control sample (Figure 5C), indicating that only Tax_WT_ induced the up-regulation of expression of this gene related to immune response/response to virus in 23CLN cells. Finally, Western blotting with polyclonal antibodies against FOS, RRAD and TNFAIP6 showed the overexpression of these proteins in the presence of Tax in 23CLN cells and noticed that FOS expression was higher in the presence of Tax_WT_ or Tax_D247G_ than compared with the presence of Tax_S240P_ and RRAD and TNFAIP6 were mainly overexpressed in the presence of Tax_D247G_ (Figure 5D). From these results, we confirmed that the regulation of genes in bovine cells expressing Tax was similar to the microarray results observed in human cells.

## Discussion

Using interesting mutant forms of the BLV Tax protein with elevated (Tax_D247G_) or reduced (Tax_S240P_) transactivation activity in the replication and propagation of BLV [14], we identified cellular genes regulated by Tax protein by a large-scale host cell gene-profiling approach using human cDNA microarrays. Our results lead to 3 major conclusions as follows. (1) Several alterations in gene expression were observed after the expression of wild-type and mutant Tax proteins, including genes involved in transcription/translation/RNA processing, signal transduction, cell growth, apoptosis, stress response, and immune response. This clearly showed that BLV Tax protein has multiple biological effects on various cellular environments via the direct or indirect interaction of Tax with cellular partners. (2) Microarray data revealed that although all Tax proteins can induce gene expression changes in HeLa cells, the number and level of changes differ among the 3 Tax proteins. Particularly, Tax_D247G_ could induce high levels and large numbers of host gene expression changes, contrary to Tax_S240P_, which induced very low levels and small numbers of gene expression changes, suggesting a correlation between gene expression and the transactivation activity of these mutants, as previously reported [8,14]. (3) The most interesting outcome of this study is the discovery that Tax and its mutants induced downregulation of expression of genes involved in the innate immune response. Moreover, genes related to the cellular stress response are also upreglated by Tax and this finding is particularly relevant to Tax_S240P._ The previously identified functions of BLV Tax are the activation of viral transcription [2,4], modulation of cellular genes related to the regulation of cell growth such as c-*fos*[8], induction of transformation and immortalization [10], regulation of apoptosis [16], inhibition of DNA repair [13] and promotion of cell survival [11,17]. Therefore, these alterations in gene expression may suggest the presence of novel functions of the BLV Tax protein that are related with immune responses and stress responses.

Previously, we identified a series of mutant BLV Tax proteins, including Tax_D247G_ and Tax_S240P_, which exhibit strikingly enhanced ability to stimulate or reduce viral LTR-directed transcription than the wild-type protein [8]. All of the mutants have at least 1 amino acid substitution between amino acids 240 and 265, and the CRE motif in the BLV LTR is sufficient for transactivation by the Tax mutants. Transient expression analysis revealed that Tax_D247G_, which had the strongest transactivation activity, more strongly increased the production of viral protein and particles from a defective recombinant proviral clone of BLV than the wild-type Tax did; conversely, Tax_S240P_ was unable to induce the release of viral particles. The present study shows that for half of the genes listed in Table 1, Tax_D247G_ induced higher gene expression than Tax_WT_ and Tax_S240P_ did, and for many of these genes, with exceptions for genes related with stress responses, were expressed at the lowest levels in Tax_S240P_-transfected cells. These results indicate that in addition to the transactivation activity of BLV LTR, the expression of cellular genes might be limited or negatively regulated by the region of the BLV Tax protein between amino acids 240 and 265 and that the substitution D247G might act to circumvent this inhibitory activity. Furthermore, Tax_D247G_, but not Tax_S240P_, was found to activate other retroviral enhancers that are not activated or are only very slightly activated by the wild-type protein [14]. Moreover, Tax_D247G_ transfection resulted in remarkably increased levels of endogenous c-*fos* mRNA in both human and bovine cell lines [8]. Conversely, the wild-type Tax protein cannot activate the expression of human c-*fos*, indicating that wild-type BLV Tax might discriminate between human and bovine c- *fos* promoter sequences. Interestingly, we found that Tax_D247G_ regulated more genes involved in transcription, signal transduction, immune responses, cell growth, apoptosis, cell adhesion, transport, metabolic processes, phosphorylation, and other functions than Tax_WT_ and Tax_S240P_ did. By contrast, the expression of heat-shock proteins (HSPs) such as DNAJB1, HSPA1A, and HSPA6, which are upregulated when cells are exposed to elevated temperatures or other stress [19], is significantly increased in the presence of Tax_S240P_ compared with that in the presence of other Tax proteins, indicating that BLV Tax may directly or indirectly be involved in stress response. These findings raised the possibility that the target sequence specificity of BLV Tax might be limited by the region of the protein between amino acids 240-265. Thus, the mutant proteins might have the ability to activate various cellular genes that might be barely activated, if at all, by wild-type Tax. Therefore, mutant forms of the BLV Tax protein, such as Tax_D247G_ and Tax_S240_, might be useful tools for elucidating the mechanism of known and novel functions induced by Tax expression. Our data also demonstrate the significant differences in the activating potential of Tax_WT_, Tax_D247G,_ and Tax_S240_, given that global changes in celluar gene expression are likely to critical to the transforming activity of these Tax mutants.

Our microarray data and qRT-PCR analysis clearly revealed that Tax transfection resulted in overexpression of FOS, and its mRNA expression level was much higher in bovine cells than in human cells. Western blotting also revealed the overexpression of FOS in the presence of Tax in both bovine 23CLN cells and HeLa cells. Furthermore, musculoaponeurotic fibrosarcoma (MAF) proteins, such as MAFB and MAFF, and JUN were significantly upregulated in the presence of Tax proteins (Table 1). Moreover, ATF-2 transcriptional factor network was significantly detected as the Tax-related signaling pathway by GeneSpring software (Table 3). It is well known that FOS, JUN, MAF, and ATF belong to the activator protein 1 (AP-1) transcription factor family [20]. AP-1 is a dimeric complex composed of JUN, FOS, MAF, and ATF protein families. This AP-1 complex can form many different combinations of heterodimers and homodimers, and different AP-1 dimers control transcriptional activation or suppression of a variety of genes involved in the regulation of proliferation, differentiation, apoptosis, and transformation. Some members of the CREB protein families are also part of AP-1 complexes. Indeed, CREM, which is member of the CREB family, and ETS oncogene transcription family member ETV5 [21], which is known to cooperate with AP-1, were upregulated by Tax protein (Table 1). As shown in Table 3, signaling pathways regulated by Tax appeared to be involved in many transcriptional factor networks related to AP-1, such as Wnt-mediated signaling, MAP kinase signaling, IL1-, IL-2-, and IL12-mediated signaling, calcium signaling, and RAS signaling. These results were supported by previous results that the expression of the FOS gene is modulated by BLV Tax protein [8,12,22], and in addition, BLV Tax protein could mediate transactivation of the viral genome through increased binding of the cellular proteins CREB, ATF-1, and ATF-2 to the TxREs [6,23]. Furthermore, results were confirmed by studies that reported that HTLV-1-Tax induced the expression of various family members of AP-1 such as c-JUN, JUN-D, c-FOS, and FRA-1 in T cells at the level of transcription through the AP-1-binding site, and upregulated genes contributed to the initial steps of the transformation process [24]. Thus, our findings raise the possibility that Tax mainly activates AP-1 signaling pathways via interactions with other transcriptional pathways, thereby stimulating the production of viral transcripts and induction of cellular genes.

The upregulation of AP-1 via direct overexpression or oncogenic RAS was found to correlate with a positive effect on cell transformation [25]. We showed that Tax protein activates nucleotide-binding proteins (G-Protein) such as GNB5 [26], GNG4 [27], RGS2 [28], and RGS16 [29] and GTP-binding proteins such as GEM [30] and RRAD [31]. Ras-related GTP-binding proteins comprise a superfamily consisting of many members that play important roles in cell proliferation and differentiation [32], cell cycle regulation [33], and glucose transport into cells [31,34]. It is known that these Ras-related GTP-binding proteins are linked with the AP-1 pathway. Interestingly, there is increasing evidence that the aberrant activity of numerous members of the Ras superfamily of small GTPases contributes to cancer growth, invasion, and metastasis [35]; as an example, RRAD overexpression in tumor tissue has been associated with poor prognosis among breast cancer patients [36]. Previously, Tax was reported to cooperate with the Ha-*ras* oncogene to induce the full transformation of cells that form tumors when it was injected into nude mice [10]. Thus, our results suggest a compromise of RAS superfamily such as GEM and RRAD expression in the mechanism leading to tumorigenesis by Tax.

Arguably the most surprising and significant finding of this study is that Tax protein transfection resulted in the up- and downregulation of genes related to the immune response, suggesting a novel function of BLV Tax protein, which can regulate immune response. In addition, all Tax variants down-regulated the expression of the T-cell specific factor GATA3, a notable difference with HTLV-1 Tax which cooperates together with ETS1/2 and GATA3 to activate the production of IL-5 [37]. The downregulated genes mainly belonged to the interferon family of anti-viral factors, such as IFI44, IFIH1, IFIT1, IFIT2, IFIT3, and ISG15. Interferons have emerged as major components of the innate immune system, and they are recognized for their antiviral function in addition to their antiproliferative and immunomodulatory effects on cells [38]. For BLV, IFNγ has mainly been detected to suppress viral replication [39-41]. In human immunodeficiency virus type 1 (HIV-1), IFNs have been implicated in blocking both early and late stages of the HIV-1 lifecycle [42,43]. IFIT1, an interferon-stimulated gene (ISG), has also been implicated in the antiviral actions of IFNs[44] against hepatitis C virus, West Nile virus, lymphocytic choriomeningitis virus, parainfluenza 1 virus, vesicular stomatitis virus, and encephalomyocarditis virus [45-47]. It has also been reported that ISG15 plays an important role in inhibiting bovine immunodeficiency virus replication in fetal bovine lung cells, and its expression has been associated with antiviral function [48]. IFIT3 was recently described as a key element of the antiproliferative and antiviral activity of IFNα [49-51]. Because these molecules have a primary role in antiviral function and we showed that they were all downregulated at similar levels by three Tax proteins in human and bovine cells except for up-regulation of IFIT3 gene by Tax_WT_ in bovine 23CLN cells, it appears that Tax suppresses the innate immune response to promote persistence and transmission of BLV. Indeed, we previously found no significant differences in the viral load and the expression of viral RNA for 28 weeks between sheep experimentally injected with BLVs that encode the wild-type and mutant Tax_D247G_ proteins, in contrast to *in vitro* that Tax_D247G_ produced more viral proteins and particles, and was transmitted very effectively [15], suggesting that Tax_WT_ and Tax_D247G_ which possess different transactivation abilities for BLV LTR and cellular proto-oncogene c-*fos*, might suppress similarly expression of genes involved in the immune response *in vivo* as well as our microarray results *,* thereby promoting expansion and infection of BLV on equal level *in vivo*.

The Tax-upregulated gene related to the immune response is TNFAIP6 (tumour necrosis factor alpha inductor protein 6; TSG-6). This gene is not constitutively expressed in healthy adult tissues, but it has been observed in physiological and pathological contexts that are associated with inflammation and tissue remodeling [52,53]; for example, TNFAIP6 has been observed in cultured human uterine cervical fibroblasts, in which it is upregulated in response to IL-1 and TNF-α [54]. In BLV replication, an important role for TNF-α has been suggested in the elimination of BLV during the early stage of infection and in the progression of disease during the lymphocytosis stage [41,55-57]. Moreover, it has clearly been demonstrated that the lack of TNF-α expression enhanced the persistence of BLV infection in TNF-α^-/-^ mice [58]. In contrast to TNFα, the role of TNFAIP6 in BLV infection is unknown. Nevertheless, as TNFAIP6 is upregulated in response to TNF-α, there is a possibility that TNFAIP6 may regulate the disease progression of BLV-induced leukemogenesis. Importantly, HTLV-1 dampens the TNFα response to promote persistence of HTLV-1 infection [59]. Moreover, TNFα shows promise as part of a combined therapy for the treatment of HTLV-1 induced adult T cell Leukemia [60]. Therefore, our data are likely to be important in understanding both BLV and HTLV-1 persistence in infected hosts and suggest possible strategies to improve therapies for BLV and HTLV-1-induced disease. Collectively, further investigation is required to define the role of Tax in immune responses and the function of the genes related to immune responses in BLV and HTLV-1 pathogeneses.

Genes that are up- or downregulated in the presence of Tax are likely to play important roles in transcription, signal transduction, cell growth, apoptosis, stress responses, and immune responses. Although these results were obtained using a human cDNA microarray system, we believe that the use of mutant Tax proteins (Tax_D247G_ and Tax_S240P_) in human cells is the best available system to begin preliminary studies on the effects of Tax on transformation during BLV and HTLV-1 infections. Indeed, although it may be noted to show the different expression patterns in several factors, such as FOS, JUN, RORA, GEM, RRAD, TNFAIP6 and IFIT3 genes in human and bovine cells, we could validate that the regulation of genes in bovine cells expressing Tax was similar to the microarray results observed in human cells in this study. However, we also assume that a number of genes is not equally expressed in different cell lines and/or *in vivo*. At this point, the extent of these effects and the manner in which they may be interrelated with the induction of BLV and HTLV-1 induced lymphoma or the regulation of disease progression are not known. A detailed study addressing the direct and indirect effects of Tax-mediated transformation is needed to gain a better understanding of the contribution of Tax to BLV and HTLV-1 pathogenesis. Our findings also lend some additional support to the hypothesis that viruses have evolved mechanisms of changing the host cellular milieu to facilitate viral replication and pathogenesis strategies that can be investigated at a mechanistic level, and our data suggest additional avenues for the development of novel antiretroviral therapeutics.

## Conclusion

By undertaking a comparative analysis of the BLV Tax protein with elevated (Tax_D247G_) or reduced (Tax_S240P_) transactivation activity, we found that Tax exerts a significant impact on cellular functions as diverse as transcription, translation, RNA processing, signal transduction, cell growth, apoptosis, stress response and immune response. Importantly, to our knowledge, our study is the first report that shows the extent to which BLV Tax regulates the innate immune response and it is likely that this tactic ensures ongoing viral replication and viral persistence even in the face of a strong host immune response. From our data, we suggest that understanding the molecular mechanisms used by BLV and HTLV-1 to thwart the immune response will lead to new avenues for the development of novel and effective antiretroviral therapeutics.

## Methods

### Cells and transfection

Human cervical HeLa and bovine lymph node 23CLN cells were maintained in DMEM medium (Invitrogen) supplemented with 10% heat-inactivated fetal bovine serum (FBS) and 100 units/mL penicillin/streptomycin (Sigma). For transient transfection, cells were transfected with Tax wild-type or mutant expression vectors by using FuGENE HD (Roche) according to the manufacturer’s instructions.

### Construction of plasmids

The Tax genes were amplified from pME18Neo encoding wild-type (Tax_WT_) and mutant (S240P (Tax_S240P_) and D247G (Tax_D247G_)) Tax proteins by using the primers BTaxF (5^′^-AACTCGAGGCCACCATGGCAAGTGTTGTTGGTTGGGGGCC-3^′^) and  BTaxR (5^′^-AAGCGCCGCTCACTTGTCGTCATCGTCTTTGTAGTCAAAAAGGCGGGAGAGCC-3^′^). The underlined sequences in the forward and reverse primers correspond to the restriction sites for *Xho*I and *Not*I, respectively, and the Flag sequence was included at the 3^′^ end of all Tax genes. PCR products were then introduced into the *Xho*I and *Not*I sites of the pCAGGS mammalian vector [61]. The pGV-BLTR reporter plasmid has been described previously [14]. The pRL-SV40 plasmid (Promega), which encodes a *Renilla* luciferase gene, was used for normalization of the efficiencies of transfection.

### Luciferase assay

HeLa and 23CLN cells were seeded into 24-well plates 24 h before transfection at a density of 1 × 10^5^ cells per well. Cells were transfected with 1 μg of each Tax vector, 0.5 μg of pGV-BLTR, and 0.5 μg of pRL-SV40 by using FuGENE HD, according to the manufacturer’s instructions. Luciferase activity was measured 24 h after transfection by using the Dual-Luciferase Reporter Assay System (Promega) according to the manufacturer’s protocol. The firefly luciferase reporter and the *Renilla* luciferase reactions were measured using a multilabel counter (Model 1420, Wallac Arvo, Perkin Elmer Life Sciences). Relative luciferase activity was calculated as firefly/ *Renilla* luciferase activity, and the luciferase activity of each construct was compared with that of the pGV-BLTR vector. All experiments were conducted in duplicate at 3 different times.

### Immunofluorescence

HeLa and 23CLN cells were seeded onto 22-mm-diameter coverslips in 24-well plates 24 h before transfection, at a density of 1 × 10^5^ cells/well. After 24 h of transfection with each Tax vector or control vector, cells were washed twice with phosphate-buffered saline (PBS), fixed in 3.7% formaldehyde, permeabilized using 0.2% Triton X-100, and stained using an anti-Flag M2 MAb (Sigma), followed by an anti-mouse IgG_1_ Alexa 488 antibody (Molecular Probes). Nuclei were stained using Hoechst 33342 (Sigma). Subcellular localization was determined using an Olympus fluorescence microscope (Olympus, FV1000).

### RNA extraction

After 24 h of transfection with each Tax vector or control vector, some cells were analyzed by Immunofluorescence. RNA was isolated from total cell extracts of HeLa or 23CLN cells by using an RNeasy Mini Kit (Qiagen) according to the manufacturer's instructions at 30 h posttransfection when the expression efficiency was greater than 30% in all transfections. For high quality of the RNA, the protocol included the remove of residual DNA in the sample. RNA was quantified using a spectrophotometer and then stored at −80°C. For gene-chip analysis, the quality of RNA was tested using the Agilent Bioanalyser (Agilent Technologies) runs.

### Microarray analysis

RNA samples were run once on microarray using the HGU133A 2.0 Affymetrix chip. Microarray hybridization and fluorescence detection were performed as described in the Affymetrix Gene-Chip Expression Analysis Technical Manual. Microarray data analyses were subjected to bioinformatics process to identify statistically significant changes in gene expression between samples by using GeneSpring GX 11.0 software (Agilent Technologies). Microarray data have been deposited in NCBI’s Gene Expression Omnibus and assigned GEO Series accession number GSE35823. We obtained fold changes in gene expression, hierarchical clustering, and gene ontology annotations, and revealed which pathways were significantly up- or downregulated (p < 0.05).

### qRT-PCR

After 30 h of transfection with each Tax vector or control vector, cells were lysed, and total RNA was prepared using the RNeasy Mini Kit (Qiagen). RT-PCR was performed using specific primers for the targets and OneStep SYBR Green PCR mix (Takara), according to the manufacturer’s manual. qRT-PCR was performed using a Prism 7500 sequence detection system (Applied Biosystems). Samples were run in duplicates and all data were normalized to GAPDH mRNA expression.

### Western blot analysis

After 30 h of transfection with each Tax vector or control vector, cells were lysed, separated through a 6–10% sodium dodecyl (SDS)-polyacrylamide gel, and then transferred to a PVDF membrane (Immobilon-P, Millipore Corp.) using a Trans-blot SD semi-dry transfer cell (Bio-Rad). After the transfer, the membranes were blocked in a 5% non-fat dry milk in PBS and 0.1% Tween-20 and then incubated with a 1:1000 dilution of primary antibody anti-Flag M2 MAb (Sigma) or a 1:300 dilution in the case of FOS (K-25), RORA (X-23), GEM (G-1), RRAD (D-15), TSG-6 (N-20), or Actin (c-11) antibodies; all antibodies were obtained from Santa Cruz Biotechnology. Thereafter, the membranes were washed and incubated either with anti-mouse, anti-rabbit, or anti-goat horseradish peroxidase-conjugated secondary antibody (Jackson, Immuno Research) and developed using the SuperSignal West Pico Chemiluminescent substrate Kit (Pierce).

## Competing interests

The authors declare that they have no competing interests.

## Authors' contributions

MA participated in all experiments, analyzed the data, and drafted the manuscript. ET participated in some experiments and analyzed some data. YA conceived the study, participated in the experiments, participated in the experimental design, coordinated the experiments, and drafted the manuscript. All authors read and approved the final manuscript.

## References

[B1] Burny A Cleuter Y Kettmann R Mammerickx M Marbaix G Portetelle D Van den Broeke A Willems L Thomas R Bovine leukemia: facts and hypotheses derived from the study of an infectious cancer Adv Vet Sci Comp Med 1988 32 149 170 2847501 10.1016/b978-0-12-039232-2.50010-4

[B2] Derse D Bovine leukemia virus transcription is controlled by a virus-encoded trans-acting factor and by cis-acting response elements J Virol 1987 61 8 2462 2471 3037109 10.1128/jvi.61.8.2462-2471.1987 PMC255671

[B3] Derse D Trans-acting regulation of bovine leukemia virus mRNA processing J Virol 1988 62 4 1115 1119 2831374 10.1128/jvi.62.4.1115-1119.1988 PMC253117

[B4] Willems L Gegonne A Chen G Burny A Kettmann R Ghysdael J The bovine leukemia virus p34 is a transactivator protein EMBO J 1987 6 11 3385 3389 2828028 10.1002/j.1460-2075.1987.tb02661.x PMC553795

[B5] Adam E Kerkhofs P Mammerickx M Burny A Kettmann R Willems L The CREB, ATF-1, and ATF-2 transcription factors from bovine leukemia virus-infected B lymphocytes activate viral expression J Virol 1996 70 3 1990 1999 8627725 10.1128/jvi.70.3.1990-1999.1996 PMC190028

[B6] Adam E Kerkhofs P Mammerickx M Kettmann R Burny A Droogmans L Willems L Involvement of the cyclic AMP-responsive element binding protein in bovine leukemia virus expression in vivo J Virol 1994 68 9 5845 5853 8057465 10.1128/jvi.68.9.5845-5853.1994 PMC236989

[B7] Boros IM Tie F Giam CZ Interaction of bovine leukemia virus transactivator Tax with bZip proteins Virology 1995 214 1 207 214 10.1006/viro.1995.9939 8525616

[B8] Tajima S Aida Y Mutant tax protein from bovine leukemia virus with enhanced ability to activate the expression of c-fos J Virol 2002 76 5 2557 2562 10.1128/jvi.76.5.2557-2562.2002 11836435 PMC135937

[B9] Kerkhofs P Heremans H Burny A Kettmann R Willems L In vitro and in vivo oncogenic potential of bovine leukemia virus G4 protein J Virol 1998 72 3 2554 2559 9499124 10.1128/jvi.72.3.2554-2559.1998 PMC109563

[B10] Willems L Heremans H Chen G Portetelle D Billiau A Burny A Kettmann R Cooperation between bovine leukaemia virus transactivator protein and Ha-ras oncogene product in cellular transformation EMBO J 1990 9 5 1577 1581 2158445 10.1002/j.1460-2075.1990.tb08277.x PMC551852

[B11] Szynal M Cleuter Y Beskorwayne T Bagnis C Van Lint C Kerkhofs P Burny A Martiat P Griebel P Van den Broeke A Disruption of B-cell homeostatic control mediated by the BLV-Tax oncoprotein: association with the upregulation of Bcl-2 and signaling through NF-kappaB Oncogene 2003 22 29 4531 4542 10.1038/sj.onc.1206546 12881710

[B12] Klener P Szynal M Cleuter Y Merimi M Duvillier H Lallemand F Bagnis C Griebel P Sotiriou C Burny A Insights into gene expression changes impacting B-cell transformation: cross-species microarray analysis of bovine leukemia virus tax-responsive genes in ovine B cells J Virol 2006 80 4 1922 1938 10.1128/JVI.80.4.1922-1938.2006 16439548 PMC1367148

[B13] Philpott SM Buehring GC Defective DNA repair in cells with human T-cell leukemia/bovine leukemia viruses: role of tax gene J Natl Cancer Inst 1999 91 11 933 942 10.1093/jnci/91.11.933 10359545

[B14] Tajima S Aida Y The region between amino acids 245 and 265 of the bovine leukemia virus (BLV) tax protein restricts transactivation not only via the BLV enhancer but also via other retrovirus enhancers J Virol 2000 74 23 10939 10949 10.1128/JVI.74.23.10939-10949.2000 11069988 PMC113173

[B15] Tajima S Takahashi M Takeshima SN Konnai S Yin SA Watarai S Tanaka Y Onuma M Okada K Aida Y A mutant form of the tax protein of bovine leukemia virus (BLV), with enhanced transactivation activity, increases expression and propagation of BLV in vitro but not in vivo J Virol 2003 77 3 1894 1903 10.1128/JVI.77.3.1894-1903.2003 12525624 PMC140974

[B16] Takahashi M Tajima S Okada K Davis WC Aida Y Involvement of bovine leukemia virus in induction and inhibition of apoptosis Microb Infect/Institut Pasteur 2005 7 1 19 28 10.1016/j.micinf.2004.09.014 15716078

[B17] Takahashi M Tajima S Takeshima SN Konnai S Yin SA Okada K Davis WC Aida Y Ex vivo survival of peripheral blood mononuclear cells in sheep induced by bovine leukemia virus (BLV) mainly occurs in CD5- B cells that express BLV Microb Infect/Institut Pasteur 2004 6 6 584 595 10.1016/j.micinf.2004.02.014 15158193

[B18] Roussev RK Shikova E Portetelle D Alexandrov I Dimitrov P Polianova M Ganchev G Expression of BLV Tax protein in lymphocytes and COS cells Exp Pathol Parasitol 2003 6 81 87

[B19] Hunt C Morimoto RI Conserved features of eukaryotic hsp70 genes revealed by comparison with the nucleotide sequence of human hsp70 Proc Natl Acad Sci U S A 1985 82 19 6455 6459 10.1073/pnas.82.19.6455 3931075 PMC390735

[B20] Wagner EF Eferl R Fos/AP-1 proteins in bone and the immune system Immunol Rev 2005 208 126 140 10.1111/j.0105-2896.2005.00332.x 16313345

[B21] Euhus DM Bu D Xie XJ Sarode V Ashfaq R Hunt KK Xia W O'Shaughnessy JA Grant M Arun BK Tamoxifen Downregulates Ets-oncogene Family Members ETV4 and ETV5 in Benign Breast Tissue: Implications for Durable Risk Reduction Cancer Prev Res (Phila) 2011 4 11 1852 1862 10.1158/1940-6207.CAPR-11-0186 21778330 PMC3208724

[B22] Katoh I Yoshinaka Y Ikawa Y Bovine leukemia virus trans-activator p38tax activates heterologous promoters with a common sequence known as a cAMP-responsive element or the binding site of a cellular transcription factor ATF EMBO J 1989 8 2 497 503 2542018 10.1002/j.1460-2075.1989.tb03403.x PMC400832

[B23] Nguyen TL Calomme C Wijmeersch G Nizet S Veithen E Portetelle D de Launoit Y Burny A Van Lint C Deacetylase inhibitors and the viral transactivator TaxBLV synergistically activate bovine leukemia virus gene expression via a cAMP-responsive element- and cAMP-responsive element-binding protein-dependent mechanism J Biol Chem 2004 279 33 35025 35036 10.1074/jbc.M404081200 15163662

[B24] Hall WW Fujii M Deregulation of cell-signaling pathways in HTLV-1 infection Oncogene 2005 24 39 5965 5975 10.1038/sj.onc.1208975 16155603

[B25] Eferl R Wagner EF AP-1: a double-edged sword in tumorigenesis Nat Rev Cancer 2003 3 11 859 868 10.1038/nrc1209 14668816

[B26] Witherow DS Slepak VZ A novel kind of G protein heterodimer: the G beta5-RGS complex Receptors Channels 2003 9 3 205 212 10.1080/10606820308239 12775340

[B27] Kalyanaraman S Copeland NG Gilbert DG Jenkins NA Gautam N Structure and chromosomal localization of mouse G protein subunit gamma 4 gene Genomics 1998 49 1 147 151 10.1006/geno.1998.5223 9570961

[B28] Kehrl JH Sinnarajah S RGS2: a multifunctional regulator of G-protein signaling Int J Biochem Cell Biol 2002 34 5 432 438 10.1016/S1357-2725(01)00141-8 11906816

[B29] Doi M Ishida A Miyake A Sato M Komatsu R Yamazaki F Kimura I Tsuchiya S Kori H Seo K Circadian regulation of intracellular G-protein signalling mediates intercellular synchrony and rhythmicity in the suprachiasmatic nucleus Nat Commun 2011 2 327 21610730 10.1038/ncomms1316 PMC3112533

[B30] Maguire J Santoro T Jensen P Siebenlist U Yewdell J Kelly K Gem: an induced, immediate early protein belonging to the Ras family Science 1994 265 5169 241 244 10.1126/science.7912851 7912851

[B31] Moyers JS Bilan PJ Reynet C Kahn CR Overexpression of Rad inhibits glucose uptake in cultured muscle and fat cells J Biol Chem 1996 271 38 23111 23116 10.1074/jbc.271.38.23111 8798502

[B32] Lange-Carter CA Johnson GL Ras-dependent growth factor regulation of MEK kinase in PC12 cells Science 1994 265 5177 1458 1461 10.1126/science.8073291 8073291

[B33] Ren M Drivas G D'Eustachio P Rush MG Ran/TC4: a small nuclear GTP-binding protein that regulates DNA synthesis J Cell Biol 1993 120 2 313 323 10.1083/jcb.120.2.313 8421051 PMC2119524

[B34] Cormont M Bortoluzzi MN Gautier N Mari M van Obberghen E Le Marchand-Brustel Y Potential role of Rab4 in the regulation of subcellular localization of Glut4 in adipocytes Mol Cell Biol 1996 16 12 6879 6886 8943343 10.1128/mcb.16.12.6879 PMC231691

[B35] Vigil D Cherfils J Rossman KL Der CJ Ras superfamily GEFs and GAPs: validated and tractable targets for cancer therapy? Nat Rev Cancer 2010 10 12 842 857 10.1038/nrc2960 21102635 PMC3124093

[B36] Tseng YH Vicent D Zhu J Niu Y Adeyinka A Moyers JS Watson PH Kahn CR Regulation of growth and tumorigenicity of breast cancer cells by the low molecular weight GTPase Rad and nm23 Cancer Res 2001 61 5 2071 2079 11280768

[B37] Blumenthal SG Aichele G Wirth T Czernilofsky AP Nordheim A Dittmer J Regulation of the human interleukin-5 promoter by Ets transcription factors. Ets1 and Ets2, but not Elf-1, cooperate with GATA3 and HTLV-I Tax1 J Biol Chem 1999 274 18 12910 12916 10.1074/jbc.274.18.12910 10212281

[B38] Hu R Gan Y Liu J Miller D Zoon KC Evidence for multiple binding sites for several components of human lymphoblastoid interferon-alpha J Biol Chem 1993 268 17 12591 12595 8509399

[B39] Keefe RG Ferrick DA Stott JL Cytokine transcription in lymph nodes of cattle in different stages of bovine leukemia virus infection Vet Immunol Immunopathol 1997 59 3–4 271 283 9477477 10.1016/s0165-2427(97)00083-4

[B40] Sentsui H Murakami K Inoshima Y Yokoyama T Inumaru S Anti-viral effect of recombinant bovine interferon gamma on bovine leukaemia virus Cytokine 2001 16 6 227 231 10.1006/cyto.2001.0967 11884026

[B41] Usui T Konnai S Ohashi K Onuma M Expression of tumor necrosis factor-alpha in IgM + B-cells from bovine leukemia virus-infected lymphocytotic sheep Vet Immunol Immunopathol 2006 112 3–4 296 301 16621026 10.1016/j.vetimm.2006.03.002

[B42] Agy MB Acker RL Sherbert CH Katze MG Interferon treatment inhibits virus replication in HIV-1- and SIV-infected CD4+ T-cell lines by distinct mechanisms: evidence for decreased stability and aberrant processing of HIV-1 proteins Virology 1995 214 2 379 386 10.1006/viro.1995.0047 8553538

[B43] Barr SD Smiley JR Bushman FD The interferon response inhibits HIV particle production by induction of TRIM22 PLoS Pathog 2008 4 2 e1000007 10.1371/journal.ppat.1000007 18389079 PMC2279259

[B44] Li Y Li C Xue P Zhong B Mao AP Ran Y Chen H Wang YY Yang F Shu HB ISG56 is a negative-feedback regulator of virus-triggered signaling and cellular antiviral response Proc Natl Acad Sci U S A 2009 106 19 7945 7950 10.1073/pnas.0900818106 19416887 PMC2683125

[B45] Guo J Peters KL Sen GC Induction of the human protein P56 by interferon, double-stranded RNA, or virus infection Virology 2000 267 2 209 219 10.1006/viro.1999.0135 10662616

[B46] Wacher C Muller M Hofer MJ Getts DR Zabaras R Ousman SS Terenzi F Sen GC King NJ Campbell IL Coordinated regulation and widespread cellular expression of interferon-stimulated genes (ISG) ISG-49, ISG-54, and ISG-56 in the central nervous system after infection with distinct viruses J Virol 2007 81 2 860 871 10.1128/JVI.01167-06 17079283 PMC1797448

[B47] Wang C Pflugheber J Sumpter R Sodora DL Hui D Sen GC Gale M Alpha interferon induces distinct translational control programs to suppress hepatitis C virus RNA replication J Virol 2003 77 7 3898 3912 10.1128/JVI.77.7.3898-3912.2003 12634350 PMC150642

[B48] Liu C Li X Yao X Kong X Qiao W Geng Y Bovine ISG15: an antiviral and inducible protein in BIV infected fetal bovine lung cells Virol J 2010 7 134 10.1186/1743-422X-7-134 20569475 PMC2900246

[B49] Lou YJ Pan XR Jia PM Li D Xiao S Zhang ZL Chen SJ Chen Z Tong JH IRF-9/STAT2 [corrected] functional interaction drives retinoic acid-induced gene G expression independently of STAT1 Cancer Res 2009 69 8 3673 3680 10.1158/0008-5472.CAN-08-4922 19351818

[B50] Schmeisser H Mejido J Balinsky CA Morrow AN Clark CR Zhao T Zoon KC Identification of alpha interferon-induced genes associated with antiviral activity in Daudi cells and characterization of IFIT3 as a novel antiviral gene J Virol 2010 84 20 10671 10680 10.1128/JVI.00818-10 20686046 PMC2950578

[B51] Xiao S Li D Zhu HQ Song MG Pan XR Jia PM Peng LL Dou AX Chen GQ Chen SJ RIG-G as a key mediator of the antiproliferative activity of interferon-related pathways through enhancing p21 and p27 proteins Proc Natl Acad Sci U S A 2006 103 44 16448 16453 10.1073/pnas.0607830103 17050680 PMC1637602

[B52] Glant TT Kamath RV Bardos T Gal I Szanto S Murad YM Sandy JD Mort JS Roughley PJ Mikecz K Cartilage-specific constitutive expression of TSG-6 protein (product of tumor necrosis factor alpha-stimulated gene 6) provides a chondroprotective, but not antiinflammatory, effect in antigen-induced arthritis Arthritis Rheum 2002 46 8 2207 2218 10.1002/art.10555 12209527

[B53] Wisniewski HG Vilcek J TSG-6: an IL-1/TNF-inducible protein with anti-inflammatory activity Cytokine Growth Factor Rev 1997 8 2 143 156 10.1016/S1359-6101(97)00008-7 9244409

[B54] Shimizu D Hosoya N Ogawa M Konishi Y Sato H Hirano H Tanaka T Expression of tumor necrosis factor-alpha stimulated gene-6 mRNA in cultured human uterine cervical fibroblasts Acta Obstet Gynecol Scand 2005 84 8 780 787 16026405 10.1111/j.0001-6349.2005.00520.x

[B55] Kabeya H Fukuda A Ohashi K Sugimoto C Onuma M Tumor necrosis factor alpha and its receptors in experimentally bovine leukemia virus-infected sheep Vet Immunol Immunopathol 2001 81 1–2 129 139 11498252 10.1016/s0165-2427(01)00338-5

[B56] Kabeya H Ohashi K Oyunbileg N Nagaoka Y Aida Y Sugimoto C Yokomizo Y Onuma M Up-regulation of tumor necrosis factor alpha mRNA is associated with bovine-leukemia virus (BLV) elimination in the early phase of infection Vet Immunol Immunopathol 1999 68 2–4 255 265 10438324 10.1016/s0165-2427(99)00029-x

[B57] Konnai S Usui T Ikeda M Kohara J Hirata T Okada K Ohashi K Onuma M Imbalance of tumor necrosis factor receptors during progression in bovine leukemia virus infection Virology 2005 339 2 239 248 10.1016/j.virol.2005.06.010 15993916

[B58] Muller C Coffey TJ Koss M Teifke JP Langhans W Werling D Lack of TNF alpha supports persistence of a plasmid encoding the bovine leukaemia virus in TNF(-/-) mice Vet Immunol Immunopathol 2003 92 1–2 15 22 12628760 10.1016/s0165-2427(03)00020-5

[B59] Yang YC Hsu TY Lin RH Su IJ Chen JY Yang CS Resistance to tumor necrosis factor-alpha-induced apoptosis in human T-lymphotropic virus type I-infected T cell lines AIDS Res Hum Retroviruses 2002 18 3 207 212 10.1089/08892220252781266 11839155

[B60] Matsuda T Almasan A Tomita M Uchihara JN Masuda M Ohshiro K Takasu N Yagita H Ohta T Mori N Resistance to Apo2 ligand (Apo2L)/tumor necrosis factor-related apoptosis-inducing ligand (TRAIL)-mediated apoptosis and constitutive expression of Apo2L/TRAIL in human T-cell leukemia virus type 1-infected T-cell lines J Virol 2005 79 3 1367 1378 10.1128/JVI.79.3.1367-1378.2005 15650163 PMC544134

[B61] Niwa H Yamamura K Miyazaki J Efficient selection for high-expression transfectants with a novel eukaryotic vector Gene 1991 108 2 193 199 10.1016/0378-1119(91)90434-D 1660837

